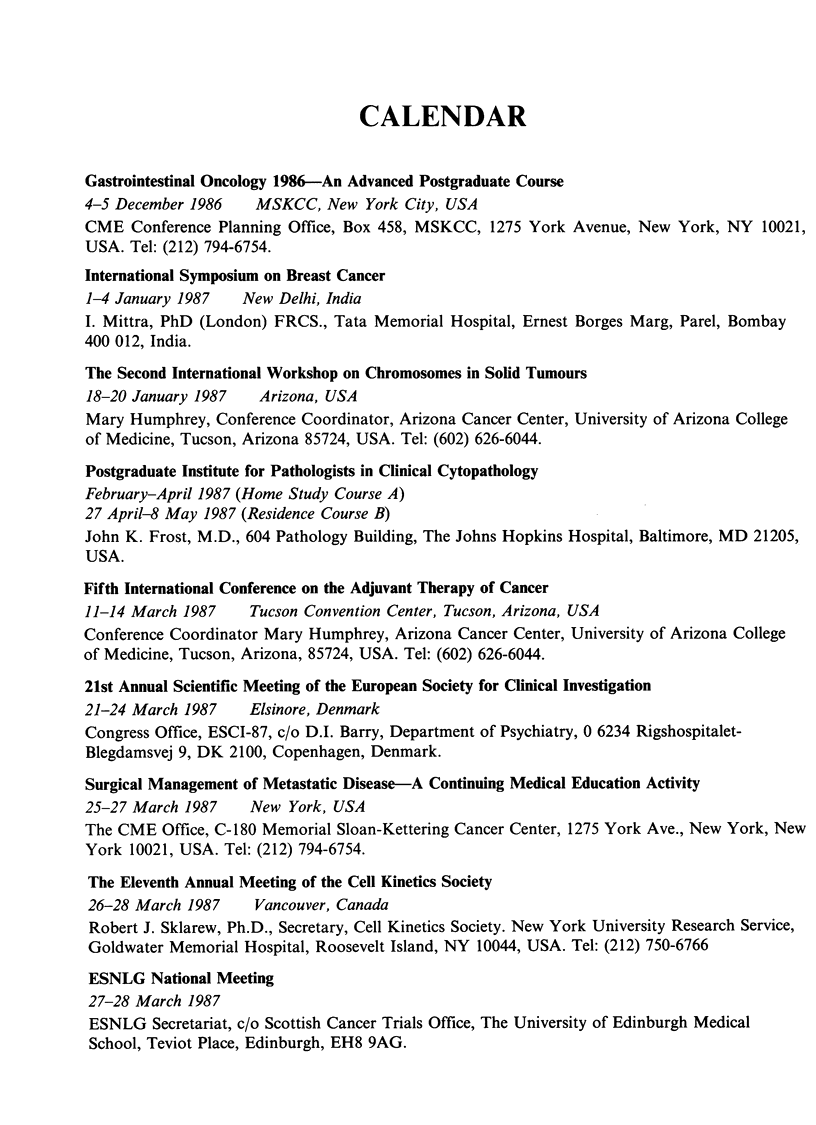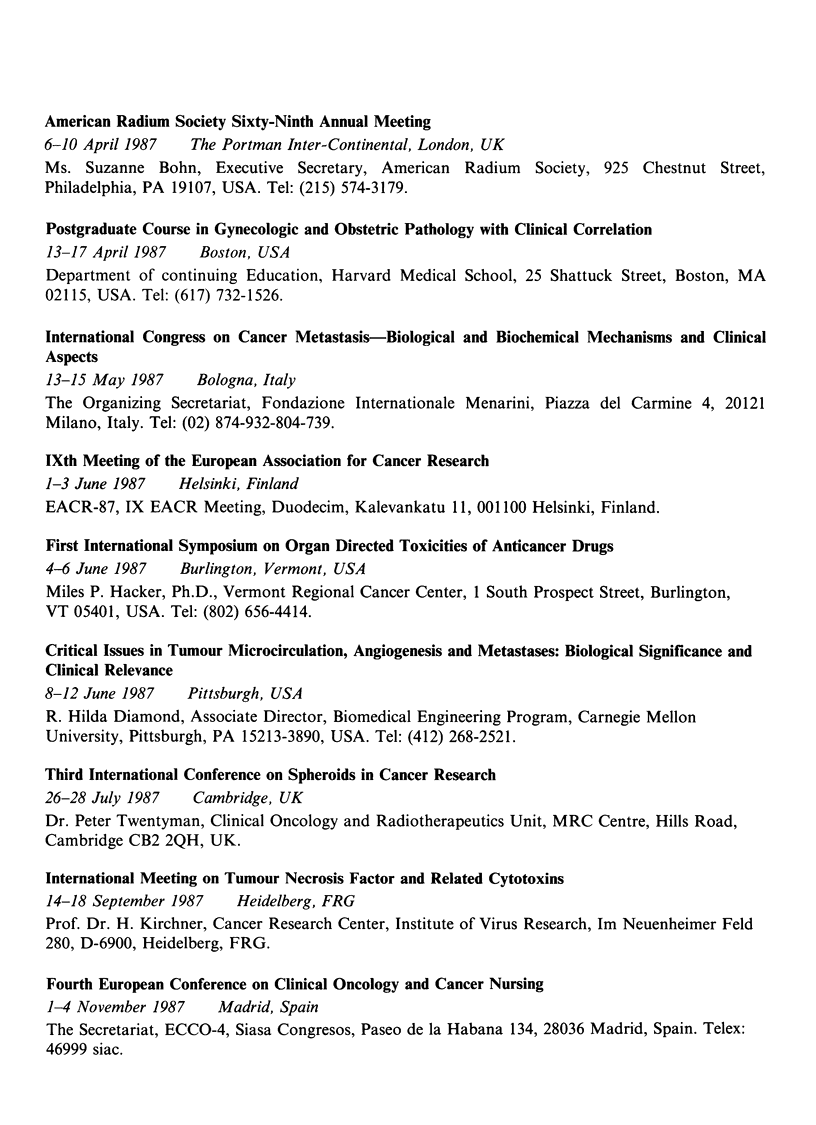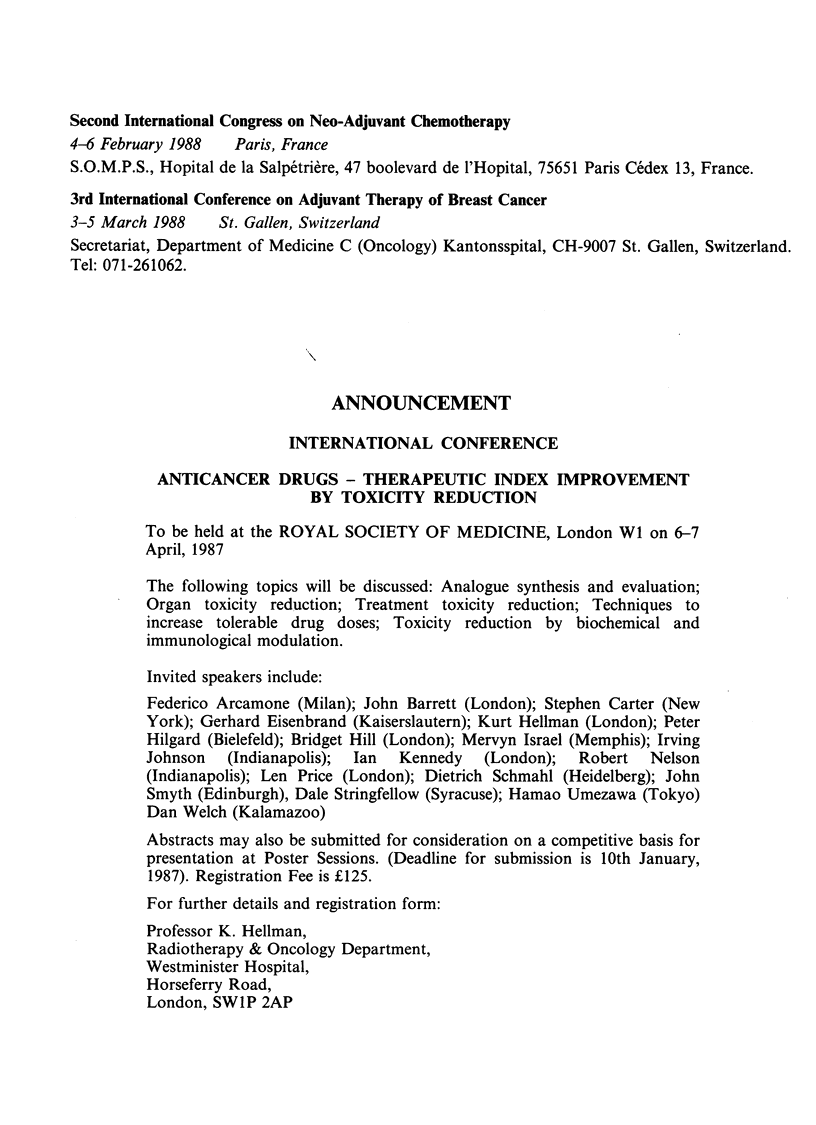# Calendar

**Published:** 1986-12

**Authors:** 


### CALENDAR

**Gastrointestinal Oncology 1986—An Advanced Postgraduate Course**
*4–5 December 1986  MSKCC, New York City, USA*
CME Conference Planning Office, Box 458, MSKCC, 1275 York Avenue, New York, NY 10021, USA. Tel: (212) 794-6754.

**International Symposium on Breast Cancer**
*1–4 January 1987  New Delhi, India*
I. Mittra, PhD (London) FRCS., Tata Memorial Hospital, Ernest Borges Marg, Parel, Bombay 400 012, India.

**The Second International Workshop on Chromosomes in Solid Tumours**
*18–20 January 1987  Arizona, USA*
Mary Humphrey, Conference Coordinator, Arizona Cancer Center, University of Arizona College of Medicine, Tucson, Arizona 85724, USA. Tel: (602) 626-6044.

**Postgraduate Institute for Pathologists in Clinical Cytopathology**
*February–April 1987 (Home Study Course A)*
*27 April–8 May 1987 (Residence Course B)*
John K. Frost, M.D., 604 Pathology Building, The Johns Hopkins Hospital, Baltimore, MD 21205, USA.

**Fifth International Conference on the Adjuvant Therapy of Cancer**
*11–14 March 1987  Tucson Convention Center, Tucson, Arizona, USA*
Conference Coordinator Mary Humphrey, Arizona Cancer Center, University of Arizona College of Medicine, Tucson, Arizona, 85724, USA. Tel: (602) 626-6044.

**21st Annual Scientific Meeting of the European Society for Clinical Investigation**
*21–24 March 1987  Elsinore, Denmark*
Congress Office, ESCI-87, c/o D.I. Barry, Department of Psychiatry, 0 6234 Rigshospitalet-Blegdamsvej 9, DK 2100, Copenhagen, Denmark.

**Surgical Management of Metastatic Disease—A Continuing Medical Education Activity**
*25–27 March 1987  New York, USA*
The CME Office, C-180 Memorial Sloan-Kettering Cancer Center, 1275 York Ave., New York, New York 10021, USA. Tel: (212) 794-6754.

**The Eleventh Annual Meeting of the Cell Kinetics Society**
*26–28 March 1987  Vancouver, Canada*
Robert J. Sklarew, Ph.D., Secretary, Cell Kinetics Society. New York University Research Service, Goldwater Memorial Hospital, Roosevelt Island, NY 10044, USA. Tel: (212) 750-6766

**ESNLG National Meeting**
*27–28 March 1987*
ESNLG Secretariat, c/o Scottish Cancer Trials Office, The University of Edinburgh Medical School, Teviot Place, Edinburgh, EH8 9AG.

**American Radium Society Sixty-Ninth Annual Meeting**

*6–10 April 1987   The Portman Inter-Continental, London, UK*

Ms. Suzanne Bohn, Executive Secretary, American Radium Society, 925 Chestnut Street, Philadelphia, PA 19107, USA. Tel: (215) 574-3179.

**Postgraduate Course in Gynecologic and Obstetric Pathology with Clinical Correlation**

*13–17 April 1987   Boston, USA*

Department of continuing Education, Harvard Medical School, 25 Shattuck Street, Boston, MA 02115, USA. Tel: (617) 732-1526.

**International Congress on Cancer Metastasis—Biological and Biochemical Mechanisms and Clinical Aspects**

*13–15 May 1987   Bologna, Italy*

The Organizing Secretariat, Fondazione Internationale Menarini, Piazza del Carmine 4, 20121 Milano, Italy. Tel: (02) 874-932-804-739.

**IXth Meeting of the European Association for Cancer Research**

*1–3 June 1987   Helsinki, Finland*

EACR-87, IX EACR Meeting, Duodecim, Kalevankatu 11, 001100 Helsinki, Finland.

**First International Symposium on Organ Directed Toxicities of Anticancer Drugs**

*4–6 June 1987   Burlington, Vermont, USA*

Miles P. Hacker, Ph.D., Vermont Regional Cancer Center, 1 South Prospect Street, Burlington, VT 05401, USA. Tel: (802) 656-4414.

**Critical Issues in Tumour Microcirculation, Angiogenesis and Metastases: Biological Significance and Clinical Relevance**

*8–12 June 1987   Pittsburgh, USA*

R. Hilda Diamond, Associate Director, Biomedical Engineering Program, Carnegie Mellon University, Pittsburgh, PA 15213-3890, USA. Tel: (412) 268-2521.

**Third International Conference on Spheroids in Cancer Research**

*26–28 July 1987   Cambridge, UK*

Dr. Peter Twentyman, Clinical Oncology and Radiotherapeutics Unit, MRC Centre, Hills Road, Cambridge CB2 2QH, UK.

**International Meeting on Tumour Necrosis Factor and Related Cytotoxins**

*14–18 September 1987   Heidelberg, FRG*

Prof. Dr. H. Kirchner, Cancer Research Center, Institute of Virus Research, Im Neuenheimer Feld 280, D-6900, Heidelberg, FRG.

**Fourth European Conference on Clinical Oncology and Cancer Nursing**

*1–4 November 1987   Madrid, Spain*

The Secretariat, ECCO-4, Siasa Congresos, Paseo de la Habana 134, 28036 Madrid, Spain. Telex: 46999 siac.

**Second International Congress on Neo-Adjuvant Chemotherapy**

*4–6 February 1988* *Paris, France*

S.O.M.P.S., Hopital de la Salpétrière, 47 boolevard de l'Hopital, 75651 Paris Cédex 13, France.

**3rd International Conference on Adjuvant Therapy of Breast Cancer**

*3–5 March 1988* *St. Gallen, Switzerland*

Secretariat, Department of Medicine C (Oncology) Kantonsspital, CH-9007 St. Gallen, Switzerland. Tel: 071-261062.